# Comparison of surgical outcomes for hip fracture between older patients with and without cancer: a propensity score matching analysis

**DOI:** 10.1038/s41598-024-54932-x

**Published:** 2024-03-05

**Authors:** Chul-Ho Kim, Kyu-pyo Kim, Ji Wan Kim

**Affiliations:** 1 Department of Orthopaedic Surgery, Asan Medical Centre, University of Ulsan College of Medicine, 88, Olympic-ro 43-gil, Songpa-gu, Seoul, 05505 Republic of Korea; 2 Department of Oncology, Asan Medical Centre, University of Ulsan College of Medicine, Seoul, 05505 Republic of Korea

**Keywords:** Cancer, Hip fracture, Complication, Mortality, Cancer, Cancer epidemiology, Metastasis, Musculoskeletal system

## Abstract

Research on the treatment outcomes and mortality of patients with cancer and hip fractures remains limited. We aimed to assess the treatment outcomes and mortality in older patients with cancer and hip fractures. We retrospectively reviewed the data of 1264 patients aged ≥ 60 years treated for hip fractures between January 2005 and April 2022. The operation time, blood transfusion-related indicators, postoperative complications, reoperation rate, length of hospital stay, admission to the intensive care unit, mortality rate, and clinical scores were compared. We also performed survival analysis. Subsequently, 1:1 propensity-score matching was performed. In the unmatched cohort, we compared 273 patients with cancer and 991 controls. The cancer group exhibited a higher incidence of pneumonia (*P* = 0.025) and higher in-hospital and 1-year follow-up mortality rates (*P* = 0.044 and *P* < 0.001, respectively). In the matched cohort, the 1-year mortality rate remained higher in the cancer group (*P* < 0.001). The control group showed a higher survival rate in both unmatched and matched cohorts (*P* < 0.001 for both). The surgical outcomes for hip fractures were comparable between patients with and without cancer. We recommend surgical treatment for hip fractures in patients with cancer.

## Introduction

Along with increased life expectancy, the incidence of cancer among older adults aged ≥ 60 years is likewise rising. Approximately 70% of patients with cancer are over the age of 65^1^. Further, the average survival rate for cancer has steadily increased. In the United States, the overall 5-year survival rate for all types of cancer has increased by approximately 23% over the past three decades^2,3^. This may be attributed to the significant advancements in cancer screening and treatment technologies.

Meanwhile, the hip fracture in the older population can cause serious medical and social issues^4^. The annual incidence of osteoporotic hip fractures is increasing due to the increased life expectancy and the resultant aging population^5^. According to the recent National Health Insurance Service data from Korea, the 1-year cumulative mortality rate among individuals aged ≥ 50 years with hip fractures was 16.0%^6^, the 20-year trends according to the United States Medicare data indicate that the 1-year mortality rate among women and men was 8.8% and 20.0%, respectively^7^.

While osteoporosis is often associated with fragility hip fractures, several other medical conditions that affect bone quality can increase the frequency of these fractures^8^. Therefore, cases of hip fractures in older adults with cancer are not uncommon.

Hip fractures in older adults typically require surgical treatment^9^ to restore their independence and facilitate engagement in activities of daily living, while also alleviating pain. However, the decision between surgical treatment and palliative care is difficult for patients with cancer having a poor general condition^10^. Research on hip fractures in the increasing population of older patients with cancer is currently lacking.

Recently, Rutenberg et al^11^. reported that the short-term treatment outcomes of hip fractures in older patients with cancer were comparable to those in the general population. However, the study had a relatively small sample size and reported short-term mortality at 1 year, indicating the need for further research.

Therefore, with the objective of investigating the treatment outcomes of hip fractures in older patients with cancer and evaluating their long-term mortality in comparison with those of controls, we conducted a large-scale propensity score (PS) matching analysis on a substantial patient cohort over a study period spanning > 13 years.

We hypothesized that there is no significant difference in the outcomes of hip fracture surgery between patients with cancer and controls despite the possibility of different survival rates due to the co-morbidity among patients with cancer. Therefore, we advocate for proactive surgical treatment whenever feasible, aiming to enhance the quality of life of patients with cancer.

## Materials and methods

### Patient selection

This retrospective study was approved by our institutional review board, and the need for written informed consent was waived. Data collection was performed in accordance with relevant guidelines and regulations of the committee at a single center.

During initial screening, we included the electronic medical records of all consecutive patients who underwent surgery for femoral neck, intertrochanteric, and subtrochanteric fractures. Of 2,066 patients, 398 had cancer. Subsequently, we included (1) data obtained between January 2005 and April 2022, (2) patients with a minimum of 1-year follow-up, and (3) individuals aged ≥ 60 years.

**Figure 1:**
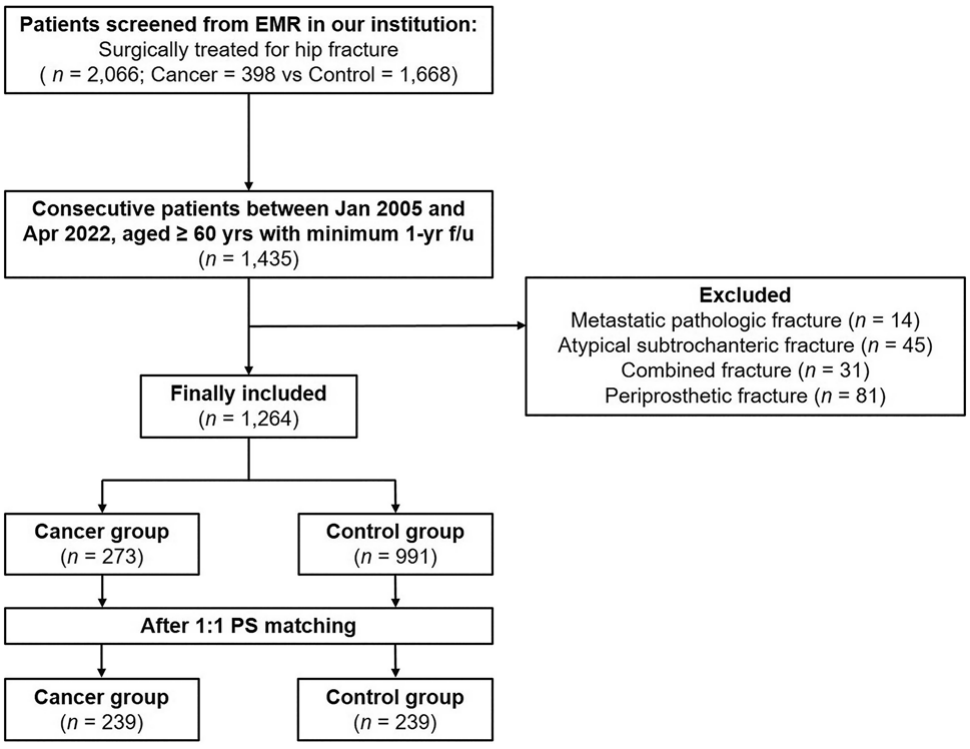
Flow chart of patient selection.

We excluded patients diagnosed with (1) metastatic pathologic fracture (*n* = 14), (2) atypical subtrochanteric femur fracture (*n* = 45), (3) concomitant fracture around the hip joint, such as a combined acetabular fracture or femoral neck and shaft fracture (*n* = 31), and (4) periprosthetic fracture (*n* = 81). The flowchart for patient selection is presented in Fig. 1.

### Study design and data collection

Initially, we identified the patients with cancer and investigated to determine the cancer type and assess the presence of cancer metastasis. A comparative analysis was conducted between the cancer group and the control group with both (1) unmatched cohorts and (2) PS matching cohorts, ensuring a balanced comparison between both groups.

We compared the following perioperative demographics of both groups: age, sex, body mass index (BMI), initial diagnosis of fracture, Charlson Comorbidity Index (CCI) score, initial Koval index, habitual status, underlying medical diseases other than cancer, time from trauma to surgery, type of surgery, and anesthesia.

We also compared parameters such as operation time, perioperative transfusion profile, perioperative medical/surgical complications, reoperation rate, hospital stay, admission status to the intensive care unit (ICU), mortality rate, and clinical scores at 1-year follow-up, including the Koval index and modified Harris hip score (mHHS).

The survival rate during follow-up in the unmatched and PS matching cohorts was compared using the Kaplan–Meier method. The endpoint was defined as patient death due to any cause.

Furthermore, we conducted a subgroup analysis comparing patients with cancer with metastasis to those without metastasis to elucidate potential differences in characteristics related to cancer severity. We also performed additional comparative analyses focusing on the three most prevalent types of cancer: gastric, lung, and colorectal cancers.

### Statistical analysis

To compare patient demographics between the cancer and control groups, the chi-square test was used for categorical variables, and the independent t-test or Wilcoxon-rank sums test was used for continuous variables. The differences between the two groups are presented as *p*-values for unmatched groups and as standardized mean differences (SMD) for the PS matched group. The SMD with a cut-off point of 0.2 was used to assess covariate balance^12^.

For the comparative analysis of outcome variables, the chi-square test or Fisher’s exact test used for categorical variables, and independent t-test or Wilcoxon-rank sum test was used for categorical variables in unmatched groups.

PSs were estimated as the probability of cancer using multiple logistic regression, incorporating all relevant demographic covariates. These covariates included patient age, sex, BMI, fracture type (diagnosis), CCI score, initial Koval index, habitual status, comorbidities other than cancer, time from trauma to surgery, type of surgery, and type of anesthesia. PS matching was performed at a ratio of 1:1 using a greedy algorithm with a caliper width of 0.2 of the standard deviation of the logit of the PS. Covariate imbalance was evaluated based on the SMD before and after PS matching. The outcomes of the PS matched set were compared through a paired t-test or Wilcoxon signed rank test for continuous variables and McNemar's test for categorical variables.

To compare the overall survival rate using the Kaplan–Meier method, the long-rank test was used for the unmatched cohort and the Prentice-Wilcoxon test for the PS matching cohort.

For the subgroup analysis, the chi-square test or Fisher’s exact test was used for categorical variables and an independent t-test or Wilcoxon-rank sums test for categorical variables.

### Ethics approval and consent to participate

This study was approved by the Institutional Review Board of Asan Medical Center and waiver was received for the need to provide written informed consent. Data collection was performed in accordance with relevant guidelines and regulations of the committee. Data cannot be shared publicly because it contains potentially identifying information of each patient. Data are available from the Asan Medical Center Institutional Data Access / Ethics Committee (contact via Asan Medical Center Institutional Review Board, Convergence Innovation Bldg. 88, Olympic-ro 43-gil, Songpa-gu, Seoul, Republic of Korea. Website link, http://eirb.amc.seoul.kr/; E-mail, irb@amc.seoul.kr; Phone, + 82–2-3010–7165).

## Results

### Study population and baseline characteristics

After applying our inclusion and exclusion criteria, 1,264 patients were enrolled in the study. Among them, 273 patients (21.6%) had a confirmed history of underlying cancer, and the remaining 991 patients were assigned to the control group. Further, 1:1 PS matching resulted in 239 matched pairs.

Gastric cancer had the highest frequency among the cancer types, accounting for 69 of the 273 patients with cancer (25.3%), followed by colorectal cancer (31/273, 11.4%) and lung cancer (30/273, 11.0%). Double primary cancer was found in 2.6% of the cases (7/273) and tumor metastasis in 19.8% (54/273). Additional details are provided in Table 1.

**Table 1:** Types of cancer in the study population.

Type of primary cancer (*n*, %)	Total (*n* = 273)
Gastric Ca	69 (25.3)
Lung Ca	30 (11.0)
Liver Ca	21 (7.7)
Colorectal Ca	31 (11.4)
Biliary Ca and Pancreatic Ca	21 (7.7)
Gynecological Ca (Uterine, Cervix, Ovary)	19 (7.0)
Breast Ca	14 (5.1)
Urologic Ca (Kidney, Ureter, Bladder)	14 (5.1)
Prostate Ca	13 (4.8)
Thyroid Ca	6 (2.2)
Hematologic Ca	16 (5.9)
Other Ca	12 (4.4)
Double primary Ca	7 (2.6)
Metastasis (*n*, %)	54 (19.8)

Ca, cancer.

### Baseline characteristics

A detailed comparison of the demographic variables of the cancer and control groups is presented in Table 2.

**Table 2:** Patients’ demographic characteristics.

	Unmatched cohort			SMD	1:1 PS matching cohort		
	Cancer patients (*n* = 273)	Control (*n* = 991)	*P*-value		Cancer patients (*n* = 239)	Control(*n* = 239)	SMD
Age, years (SD)	77.3 (8.3)	78.9 (8.2)	**0.005**	0.192	77.9 (8.3)	78.3 (8.1)	0.049
Male sex, *n* (%)	117 (42.9)	224 (22.6)	** < 0.001**	**0.442**	90 (37.7)	95 (39.7)	0.043
BMI, kg/m^2^ (SD)	21.8 (3.5)	22.4 (3.6)	**0.044**	0.140	22.0 (3.5)	21.8 (3.4)	0.040
Diagnosis, *n* (%)			0.168	0.137			0.052
Femoral neck fracture	129 (47.3)	427 (43.1)			115 (48.1)	110 (46.0)	
Intertrochanteric fracture	136 (49.8)	511 (51.6)			116 (48.5)	122 (51.0)	
Subtrochanteric fracture	8 (2.9)	53 (5.3)			8 (3.3)	7 (2.9)	
CCI score, *n* (%)			** < 0.001**	**1.039**			0.053
0–3	2 (0.7)	58 (5.9)			2 (0.8)	1 (0.4)	
4–5	24 (8.8)	458 (46.2)			24 (10.0)	24 (10.0)	
≥ 6	247 (90.5)	475 (47.9)			213 (89.1)	214 (89.5)	
Preoperative Koval index, median (IQR)	1 (1–4)	1 (1–3)	0.154	0.099	1 (1–4)	1 (1–3)	0.021
Preoperative habitual status, *n* (%)			0.306	0.101			0.049
Home	232 (85.0)	872 (88.0)			208 (87.0)	206 (86.2)	
Facility	21 (7.7)	53 (5.3)			17 (7.1)	20 (8.4)	
Unknown	20 (7.3)	66 (6.7)			14 (5.9)	13 (5.4)	
Comorbidity, *n* (%)							
DM	106 (38.8)	337 (34.0)	0.139	0.100	98 (41.0)	108 (45.2)	0.085
HTN	166 (60.8)	663 (66.9)	0.061	0.127	152 (63.6)	153 (64.0)	0.009
Stroke	35 (12.8)	167 (16.9)	0.108	0.114	35 (14.6)	31 (13.0)	0.049
DVT	7 (2.6)	24 (2.4)	0.893	0.009	7 (2.9)	5 (2.1)	0.054
Dementia	18 (6.6)	91 (9.2)	0.177	0.096	18 (7.5)	24 (10.0)	0.089
Time from trauma to surgery, days (SD)	4.1 (4.0)	3.9 (4.0)	0.485	0.058	5.1 (8.2)	4.7 (6.1)	0.067
Type of surgery, *n* (%)			0.265	0.076			0.052
Osteosynthesis	168 (61.5)	646 (65.2)			144 (60.3)	150 (62.8)	
Arthroplasty	105 (38.5)	345 (34.8)			95 (39.7)	89 (37.2)	
Anesthesia, *n* (%)			**0.004**	0.199			0.008
General	122 (44.7)	541 (54.6)			112 (46.9)	113 (47.3)	
Regional	151 (55.3)	450 (45.4)			127 (53.1)	126 (52.7)	

BMD, body mass index; BPHA, bipolar hemiarthroplasty; CCI, Charson Comorbidity Index; CCS, cannulated cancellous screw fixation; DHS, dynamic hip screw; DM, diabetes mellitus; DVT, deep vein thrombosis; FNS, femoral neck system; HTN, hypertension; IQR, interquartile range; PS, propensity score; SD, standard deviation; SMD, standard mean difference; THA, total hip arthroplasty.

Significant values are in [bold].

In the unmatched cohort, notable differences were observed between the cancer and control groups. Patients in the control group were significantly older (*P* = 0.005), and the proportion of male patients was approximately twice that in the cancer group (*P* < 0.001). The cancer group exhibited lower BMI values (*P* = 0.044) and higher CCI scores. Additionally, the frequency of regional anesthesia was significantly higher in the cancer group compared to the general anesthesia group (*P* = 0.004).

Following 1:1 PS matching, all baseline demographics were successfully matched and well-balanced, with the SMD below the cut-off point of 0.2.

### Outcome variables

A detailed comparison of the perioperative outcome variables between the cancer and control groups is shown in Table 3.

**Table 3:** Comparison between the cancer and control groups.

	Unmatched cohort			1:1 PS matching cohort		
	Cancer patients (*n* = 273)	Control (*n* = 991)	*P*-value	Cancer patients (*n* = 239)	Control (*n* = 239)	*P*-value
Operative time, min (SD)	60.9 (26.9)	66.6 (27.8)	**0.003**	62.2 (26.8)	61.5 (25.5)	0.743
Transfusion profile						
Preoperative transfusion, *n* (%)	51 (18.7)	108 (10.9)	**0.001**	44 (18.4)	32 (13.4)	0.157
Intraoperative transfusion, *n* (%)	28 (10.3)	188 (19.0)	**0.001**	24 (10.0)	32 (13.4)	0.267
Postoperative transfusion, *n* (%)	122 (44.7)	420 (42.4)	0.495	110 (46.0)	116 (48.5)	0.577
Total volume of perioperative transfusion, pack (SD)	2.0 (4.2)	1.7 (2.7)	0.846	1.9 (4.0)	1.9 (3.4)	0.839
Complications, *n* (%)						
Pneumonia	25 (9.2)	54 (5.4)	**0.025**	23 (9.6)	23 (9.6)	1.000
DVT	4 (1.5)	17 (1.7)	1.000	3 (1.3)	4 (1.7)	0.705
PTE	1 (0.4)	11 (1.1)	0.480	0 (0.0)	4 (1.7)	**0.046**
MI/Angina	6 (2.2)	18 (1.8)	0.683	5 (2.1)	6 (2.5)	0.763
UTI	9 (3.3)	47 (4.7)	0.304	8 (3.3)	10 (4.2)	0.637
Acute PJI	4 (1.5)	15 (1.5)	1.000	4 (1.7)	5 (2.1)	0.739
Rate of reoperation due to any cause, n (%)	8 (2.9)	57 (5.8)	0.062	7 (2.9)	14 (5.9)	0.127
Hospital stay, days (SD)	13.3 (11.7)	13.0 (11.1)	0.441	13.3 (11.3)	13.8 (11.2)	0.638
ICU admission, *n* (%)	47 (17.2)	145 (14.6)	0.292	41 (17.2)	43 (18.0)	0.814
Mortality rate, *n* (%)						
Death during the admission period	5 (1.8)	5 (0.5)	**0.044**	3 (1.3)	4 (1.7)	0.705
30-day mortality	9 (3.3)	14 (1.4)	0.068	5 (2.1)	7 (2.9)	0.564
1-year mortality	76 (27.8)	86 (8.7)	** < 0.001**	63 (26.4)	29 (12.1)	** < 0.001**
Clinical Score						
Koval index at the 1-year follow-up, median (IQR)	2 (1–3)	2 (1–3)	0.633	2 (1–4)	2 (1–2)	0.377
mHHS at the 1-year follow-up, point (SD)	74.9 (23.8)	73.4 (18.6)	0.566	N/A	N/A	N/A

DVT; deep vein thrombosis; ICU, intensive care unit; IQR, interquartile range; mHHS; modified Harris hip score; MI; myocardial infarction; N/A, non applicable; Op., operation; PJI, prosthetic joint infection; PS, propensity score; PTE, pulmonary thromboembolism; SD, standard deviation; UTI, urinary tract infection.

Significant values are in [bold].

In the unmatched analysis, the operation time was longer in the control group than in the cancer group (*P* = 0.003). The preoperative transfusion rate was higher in the cancer group (*P* = 0.001), while intraoperative transfusion was more common in the control group (*P* = 0.001). However, there were no significant difference in the postoperative transfusion rate or total volume of perioperative transfusion between both groups.

Pneumonia was the only postoperative complication that exhibited a significant difference between the two groups. The frequency of pneumonia in the cancer group was approximately twice that in the control group (*P* = 0.025). There was no notable difference in the reoperation rate, hospital stay, or ICU admission rate between the groups.

The mortality rates during admission and at 1-year were higher in the cancer group than in the control group (*P* = 0.044, *P* < 0.001, respectively), but there was no significant difference in the 30-day mortality rate of the two groups. Moreover, the clinical scores, including the Koval index and mHHS, showed no significant difference between both groups.

In contrast, after PS matching, no difference was observed in the operation time or transfusion profile between the two groups. The rate of postoperative pneumonia showed no difference between two groups; however, the rate of pulmonary thromboembolism was found to be significantly higher in the control group (*P* = 0.046). There was no difference in the mortality rate of both groups during the admission period, but the 1-year mortality rate was higher in the cancer group (*P* < 0.001). This finding remained consistent in the PS matched cohort. No other variables showed statistically significant differences between the two groups.

### Survival analysis

In the overall unmatched cohort, the median survival time was calculated to be 6.5 years (95% confidence interval [CI], 5.9–7.1); the cancer and control groups showed a median survival time of 2.8 years (95% CI, 2.1–3.8) and 7.4 years (95% CI, 6.7–8.4), respectively.

In the PS matched cohort, the overall median survival time was determined to be 4.3 years (95% CI, 3.6–5.0); the cancer and control groups showed a median survival time of 3.4 years (95% CI, 2.2–4.3) and 5.5 years (95% CI, 4.2–6.3), respectively.

**Figure 2:**
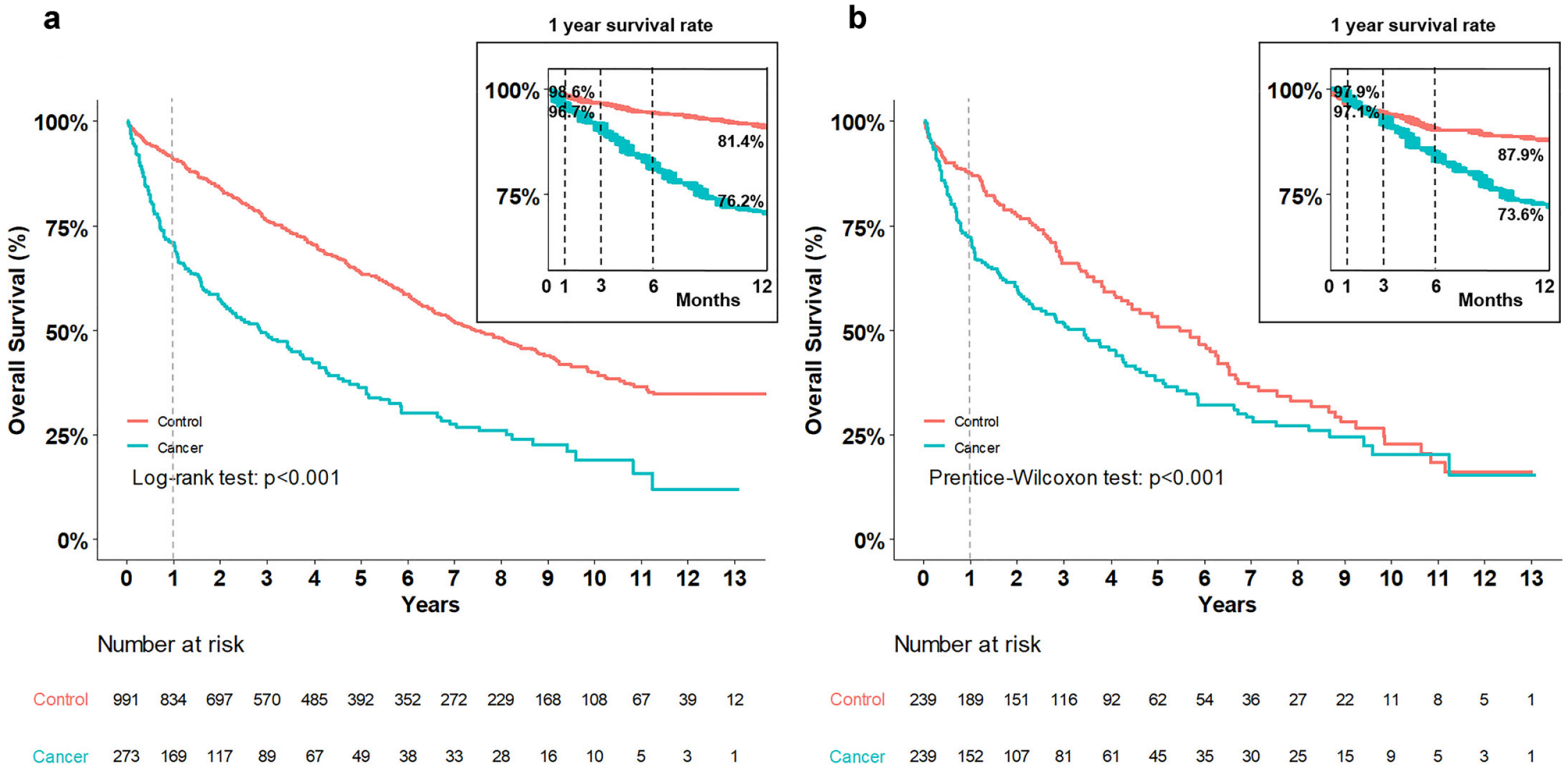
Kaplan–Meier (KM) survival analysis between the cancer and control groups. (**a**) KM curve in the unmatched cohort and (**b**) K-M curve in the 1:1 propensity matched cohort.

Figure 2 presents the Kaplan–Meier survival curve, depicting the survival outcomes in both unmatched and PS matched cohorts. During the follow-up, it was evident that the cancer group had a lower survival rate than the control group in both cohorts. Notably, the gap between the survival curves appeared to be narrower in the PS matched cohort. Further, in both unmatched and PS matched cohorts, the overall survival rate was significantly higher in the control group than in the cancer group (unmatched cohort: *P* < 0.001, and PS matched cohort: *P* < 0.001, respectively).

### Subgroup analysis

Of the 273 patients with cancer, 54 (19.8%) were confirmed to have metastasis. The demographics of patients with and without metastasis are summarized in Table 4. The most relevant location of metastasis was multiple-site metastasis, observed in 27 out of 54 cases (50%). Liver metastasis followed in 10 out of 54 cases (18.5%), while lung metastasis was observed in 6 of 54 cases (11.1%).

**Table 4:** Demographic characteristics of patients with cancer with and without metastasis.

	Metastasis ( +) (*n* = 54)	Metastasis (−) (*n* = 219)	*P*-value
Age, years (SD)	74.4 (8.7)	78.0 (8.1)	**0.005**
Male sex, *n* (%)	24 (44.4)	93 (42.5)	0.878
BMI, kg/m^2^ (SD)	22.1 (3.0)	21.7 (3.6)	0.479
Diagnosis, *n* (%)			0.348
Femoral neck fracture	25 (46.3)	104 (47.5)	
Intertrochanteric fracture	29 (53.7)	107 (48.9)	
Subtrochanteric fracture	0 (0)	8 (3.7)	
CCI score, *n* (%)			**0.011**
0–3	0 (0)	2 (0.9)	
4–5	0 (0)	24 (11.0)	
≥ 6	54 (100)	193 (88.1)	
Preoperative Koval index, median (IQR)	1 (1–3)	2 (1–4)	0.439
Preoperative habitual status, *n* (%)			0.396
Home	46 (88.5)	186 (92.5)	
Facility	6 (11.5)	15 (7.5)	
Underlying Dz., *n* (%)			
DM	17 (31.5)	89 (40.6)	0.275
HTN	28 (51.9)	138 (63.0)	0.161
Stroke	1 (1.9)	34 (15.5)	0.011
DVT	3 (5.6)	4 (1.8)	0.142
Dementia	1 (1.9)	17 (7.8)	0.215
Time from trauma to surgery, days (SD)	4.9 (6.2)	4.7 (7.3)	0.880
Type of surgery (grouping), *n* (%)			0.875
Osteosynthesis	33 (61.1)	138 (63.0)	
Arthroplasty	21 (38.9)	81 (37.0)	
Anesthesia, *n* (%)			1.000
General	24 (44.4)	98 (44.7)	
Regional	30 (55.6)	121 (55.3)	

BMI, body mass index; BPHA, bipolar hemiarthroplasty; CCI, Charson Comorbidity Index; CCS, cannulated cancellous screw fixation; DHS, dynamic hip screw; DM, diabetes mellitus; DVT, deep vein thrombosis; FNS, femoral neck system; HTN, hypertension; IQR, interquartile range; PS, propensity score; SD, standard deviation; SMD, standard mean difference; THA, total hip arthroplasty.

Significant values are in [bold].

The mean patient age and CCI score were greater in the metastasis group (*P* = 0.005 and *P* = 0.011, respectively). However, no significant differences were observed between the two groups of other patient demographics.

Among outcome variables, the 1-year mortality rate was significantly higher in the metastasis group than in the group without metastasis (*P* < 0.001). However, the other outcome variables showed no differences between groups. Additional details are provided in Table 5.

**Table 5:** Comparison of patients with cancer with and without metastasis.

	Metastasis ( +) (*n* = 54)	Metastasis (−) (*n* = 219)	*P*-value
Operative time, min (SD)	62.3 (26.8)	60.6 (27.0)	0.667
Transfusion profile			
Preoperative transfusion, *n* (%)	14 (25.9)	37 (16.9)	0.171
Intraoperative transfusion, *n* (%)	8 (14.8)	20 (9.1)	0.315
Postoperative transfusion, *n* (%)	23 (42.6)	99 (45.2)	0.762
Total volume of perioperative transfusion, pack (range)	2.2 (3.5)	2.0 (4.3)	0.706
Complications, *n* (%)			
Pneumonia	5 (9.3)	20 (9.1)	1.000
DVT	1 (0.8)	3 (1.4)	1.000
PTE	0 (0)	1 (0.5)	1.000
MI/Angina	2 (3.7)	4 (1.8)	0.339
UTI	0 (0)	9 (4.1)	0.212
Acute PJI	1 (1.9)	3 (1.4)	1.000
Rate of reoperation due to any cause, *n* (%)	1 (1.9)	7 (3.2)	1.000
Hospital stay, days (SD)	14.8 (13.6)	12.9 (11.2)	0.290
ICU admission, *n* (%)	9 (16.7)	38 (17.4)	1.000
Mortality rate, *n* (%)			
Death during the admission period	1 (1.9)	4 (1.8)	1.000
30-day mortality	3 (5.8)	5 (2.4)	0.199
1-year mortality	21 (55.3)	26 (16.5)	** < 0.001**

DVT; deep vein thrombosis; ICU, intensive care unit; IQR, interquartile range; mHHS; modified Harris hip score; MI; myocardial infarction; Op., operation; PJI, prosthetic joint infection; PS, propensity score; PTE, pulmonary thromboembolism; SD, standard deviation; UTI, urinary tract infection.

Significant values are in [bold].

The comparative analysis for the three most common cancers, namely gastric cancer, lung cancer, and colorectal cancer, is presented in Appendix 1. Consistent with the general trends, no significant differences were observed between the cancer and control groups. However, among patients with gastric cancer, the 1-year mortality rate was unexpectedly higher in the control group (*P* = 0.040), and the incidence of postoperative pneumonia was significantly higher in the lung cancer group (*P* = 0.006).

## Discussion

The principal finding of our PS matching study was that no differences were observed in the treatment outcomes and perioperative complications of hip fracture surgery between older patients with cancer and controls. Despite the higher 1-year mortality and lower survival rate in the cancer group than in the control group, the overall treatment outcomes were comparable. Furthermore, our subgroup analysis revealed that the cancer metastasis group had a significantly higher 1-year mortality rate than the group with cancer without metastasis. Therefore, it is advisable for a patient with cancer diagnosed with a hip fracture to actively consider surgical treatment, particularly in cases of cancer without metastasis. Healthcare professionals should more favorably recommend surgical intervention in these cases as it can significantly contribute to better outcomes and improved well-being and quality of life of cancer patients with hip fractures.

In the present study, the unmatched cohort analysis revealed that the cancer group had a relatively lower mean age, higher proportion of males, and lower BMI than those in the control group. In general, fragility hip fractures are more common in the older population, particularly among postmenopausal women with a high prevalence of osteoporosis^13,14^. However, in special groups, such as patients with cancer, hip fractures can occur at a relatively young age or more frequently among men than in the general population. Further, these fractures are more likely to occur in patients with cachexia, a condition often associated with cancer. Therefore, our findings align with this context and support the understanding of the unique characteristics of hip fractures in patients with cancer. Moreover, the higher CCI scores in the cancer group as compared to those in the control group, which indicate the adjustment of hip fracture mortality among older adults, can also be interpreted within the same context. The presence of cancer and its associated comorbidities can contribute to higher CCI scores.

The type of anesthesia also differed between the cancer and control groups in the unmatched cohort. This discrepancy may be attributed to the higher prevalence of cardiopulmonary comorbidities in the cancer group, which may resulted in a preference for regional anesthesia over general anesthesia in this group.

After PS matching, all variables listed in Table 2 were successfully balanced, with an SMD below the recommended threshold of 0.2. This indicates that the PS matching method was effectively applied to obtain a well-matched cohort. The balanced distribution of the variables ensured a more reliable and accurate comparison between the cancer and control groups, enhancing the validity of our findings.

Considering the perioperative outcomes, in the unmatched cohort, the operation time of the control group was more than that of the cancer group. We attribute this discrepancy to the difference in the types of surgery performed in both groups. Certain procedures, such as total hip arthroplasty or dynamic hip screw, typically require more time than other surgeries, such as bipolar hemiarthroplasty or nailing^15^. In the present study, dynamic hip screw operation and total hip arthroplasty were relatively more common in the control group of the unmatched cohort, while cephalomedullary nailing surgery and bipolar hemiarthroplasty were relatively less common. Therefore, the difference in the operation time between the groups may not have significant clinical implications as it may be attributable to variations in surgical procedures rather than any inherent difference between the cancer and control groups.

Preoperative transfusion was more frequent in the cancer group, which can be attributed to the higher burden of comorbidity typically seen in patients with cancer. These comorbidities may increase the likelihood of preoperative anemia requiring correction through transfusion. However, intraoperative transfusion rates were higher in the control group, resulting in comparable total blood transfusion volumes between the two groups. These findings suggest that regardless of cancer status, a certain amount of bleeding is inevitable on hip fracture and during its surgical treatment.

Considering perioperative complications, pneumonia occurred more frequently in the cancer group in the unmatched cohort, which is consistent with the findings of some previous studies. In their single-center retrospective study, Karam et al^16^. observed a high incidence of respiratory complications in patients with cancer undergoing total hip and knee arthroplasty. Similarly, a single-center retrospective study by Zhao et al^17^. reported that existing medical conditions, including cancer, were potential risk factors for in-hospital postoperative pneumonia following geriatric intertrochanter fracture. However, there are conflicting findings in this regard. In a recent meta-analysis on risk factors for postoperative pneumonia in patients undergoing hip fracture surgery risk by Han et al^18^., a pooled analysis of three studies did not identify cancer as a significant risk factor for postoperative pneumonia (*P* = 0.09). Consistent with this, the present findings after PS matching showed no significant difference in the incidence of postoperative pneumonia between the cancer and control groups. A previous study conducted at our institution investigated the risk factors for postoperative pneumonia and identified several significant predictors^19^, including high CCI scores, postoperative delirium, and American Society of Anesthesiologist scores ≧3.

After PS matching, we found no significant differences in the operation time and transfusion profile between the cancer and control groups. However, the incidence of pulmonary thromboembolism was higher in the control group. These findings differ from those of previous population-based studies that identified malignancy as a predictor of venous or pulmonary thromboembolism. White et al^20^. reported that malignancy was a predictor of venous or pulmonary thromboembolism (odds ratio = 1.7; 95% CI, 1.6–1.8) in emergency surgical procedures. Heit et al^21^. identified malignancy as an individual risk factor for deep vein thrombosis and pulmonary embolism. To explain these contrasting results, we propose a hypothesis. The discrepancy may be attributable to the thromboprophylaxis protocol followed at our institution. Before September 2017, our institution utilized a conventional thromboprophylaxis protocol for patients with hip fracture, which primarily involved the use of anti-embolism stockings^22^. However, a more intensive thromboprophylaxis protocol, incorporating both mechanical and chemical prophylaxis, was implemented starting October 2017. Notably, the cases of pulmonary thromboembolism identified in this study after PS matching had occurred prior to 2017, suggesting a potential bias due to the difference in protocols.

Based on the available data, no significant difference in the clinical scores, specifically the Koval index and mHHS, was identified between the cancer and control groups in both unmatched and PS-matched cohorts in the present study. Although a comparative analysis for the mHHS in the PS-matched cohort was not performed owing to missing data, it can be inferred that the functional outcomes after hip fracture surgery in the cancer group are comparable to those in the control group. Therefore, it is reasonable to conclude that the presence of cancer should not discourage the consideration of surgery for hip fractures.

In the unmatched analysis, the cancer group exhibited a higher in-hospital mortality rate than that of the control group. However, following PS matching, there was no significant difference in the in-hospital mortality between the cancer and control groups. Both unmatched and PS-matched cohorts showed no difference in the 30-day mortality rate between the groups. However, the 1-year mortality rate was significantly higher in the cancer group than in the control group in both unmatched and PS-matched cohorts. These findings suggest that while perioperative or short-term mortality rates are similar between the two groups, patients with cancer have a higher risk of mortality within the first year following hip fracture surgery. These results are consistent with the recent findings of Rutenberg et al^11^. They reported no short-term harmful effects of surgical treatment for fragility hip fractures in patients with cancer, with no difference in the in-hospital mortality of the cancer and control groups. However, they did observe a higher 1-year mortality rate in the cancer group, which they attributed to the significant comorbidity burden. In our study, we aimed to build upon these findings through a long-term follow-up study spanning over 13 years and including a larger sample. Additionally, we implemented PS matching to adjust for any potential selection bias. Our survival analysis confirmed that the control group consistently exhibited higher survival rates throughout the follow-up. However, it is noteworthy that the difference between the two groups decreased over time. Further, we did not observe significant differences in other complications between the two groups. Based on these results, the difference in the survival rates of the groups was likely influenced by cancer severity and comorbidities than surgical factors.

Our subgroup analysis comparing patients with cancer with and without metastasis led to an interesting observation. Patients with metastasis had a significantly higher 1-year mortality rate, which was more than three times higher than that in the cancer group without metastasis. Specifically, the 1-year mortality rates in cancer groups with and without metastasis were 55.3% and 16.5%, respectively. In contrast, the cancer group without metastasis exhibited characteristics similar to those of the control group. These findings suggest that metastasis can significantly influence the mortality outcomes of patients with cancer having hip fractures. The metastatic status should therefore be considered when determining the prognosis and treatment options for these patients.

The importance of surgical treatment for hip fractures in older patients has gained consensus among researchers and healthcare professionals. Numerous studies have consistently demonstrated poor outcomes associated with conservative management of hip fractures. Supporting this notion, a 2021 study by Broekman et al^23^. in the Netherlands revealed alarming results. Of 23 frail nursing home residents with hip fractures, a staggering 87% (20 of the 23) died within 1 month of receiving non-surgical treatment.

Similarly, a 2021 study conducted by Kanazawa^10^ in Japan highlighted the grim prognosis of patients with terminal cancer who experienced femoral pathologic fractures and opted for palliative care instead of surgery. These findings underscore the critical importance of surgical intervention for hip fractures in patients with cancer. Therefore, specifically in cases without metastasis and with low-grade cancer, the authors strongly recommend surgical treatment for hip fractures to improve patient outcomes and overall prognosis.

This study had several limitations. First, its retrospective design has inherent limitations; however, this study represents the first PS matching analysis in a large study population, which strengthened our findings. Additionally, the application of PS matching provided epistemological advantages over conventional regression modeling in analyzing non-randomized intervention trials^24^. Second, the missing clinical score data posed a challenge during analysis. Unfortunately, we were unable to compare the mHHS between the cancer and control groups after PS matching due to missing data. This limitation highlights the need for future studies to address and minimize missing data to obtain more comprehensive results. In cases where cancer coincides with aging-related hip fractures, simplifying treatment is not feasible, necessitating individualized care tailored to each patient's circumstances. Multidisciplinary diagnosis and treatment are required to thoroughly evaluate the patient's condition and formulate personalized treatment decisions. Therefore, despite these limitations, this study provides valuable insights into the treatment outcomes of hip fractures in patients with cancer. Future research addressing the limitations of the present study can provide more robust evidence and refine our understanding of this topic.

## Conclusion

The findings of the present study indicate that surgical intervention for hip fracture yields comparable clinical outcomes in patients with and without cancer. Therefore, we recommend considering surgical treatment of hip fractures in patients with cancer.

### Supplementary Information

Supplementary Tables.

## Data Availability

The datasets used and/or analyzed during the current study are available from the corresponding author on reasonable request.
