# Supplementary material for: Comparison of surgical outcomes for hip fracture between older patients with and without cancer: a propensity score matching analysis
Source: Sci Rep. 2024 Mar 5;14:5406. doi: 10.1038/s41598-024-54932-x (PMC10914780; doi:10.1038/s41598-024-54932-x)
Supplement: Supplementary file 1 — Supplementary Tables. [file 41598_2024_54932_MOESM1_ESM.docx]

**Appendix 1.**

**Comparison between gastric cancer patients and non-cancer patients**

|  | **Gastric Cancer** (n=69) | **Control** (n=991) | **P-value** |
| --- | --- | --- | --- |
| **Operative time, min (SD)** | 63.4 (27.9) | 66.6 (27.8) | 0.356 |
| **Transfusion profile** |  |  |  |
| Preoperative transfusion, n (%) | 13 (18.8) | 108 (10.9) | 0.051 |
| Intraoperative transfusion, n (%) | 11 (15.9) | 188 (19.0) | 0.633 |
| Postoperative transfusion, n (%) | 31 (44.9) | 420 (42.4) | 0.707 |
| Total volume of perioperative transfusion, pack (range) | 2.5 (5.3) | 1.7 (2.7) | 0.230 |
| **Complications, n (%)** |  |  |  |
| Pneumonia | 6 (8.7) | 54 (5.4) | 0.274 |
| DVT | 0 (0) | 17 (1.7) | 0.620 |
| PTE | 0 (0) | 11 (1.1) | 1.000 |
| MI/Angina | 3 (4.3) | 18 (1.8) | 0.152 |
| UTI | 1 (1.4) | 47 (4.7) | 0.362 |
| Acute PJI | 0 (0) | 15 (1.5) | 0.617 |
| **Reoperation rate for any reason, n (%)** | 2 (2.9) | 57 (5.8) | 0.424 |
| **Hospital stay, days (SD)** | 12.3 (8.4) | 13.0 (11.1) | 0.608 |
| **ICU admission, n (%)** | 15 (21.7) | 145 (14.6) | 0.118 |
| **Mortality rate,** **n (%)** |  |  |  |
| Death during the admission period | 2 (2.9) | 5 (0.5) | 0.071 |
| 30-day mortality | 2 (2.9) | 14 (1.4) | 0.239 |
| 1-year mortality | 1 (1.4) | 86 (8.7) | **0.040** |

DVT; deep vein thrombosis; ICU, intensive care unit; IQR, interquartile range; mHHS; modified Harris hip score; MI; myocardial infarction; Op., operation; PJI, prosthetic joint infection; PS, propensity score; PTE, pulmonary thromboembolism; SD, standard deviation; UTI, urinary tract infection

**Comparison between lung cancer patients and non-cancer patients**

|  | **Lung Cancer** (n=30) | **Control** (n=991) | **P-value** |
| --- | --- | --- | --- |
| **Operative time, min (SD)** | 63.5 (27.3) | 66.6 (27.8) | 0.553 |
| **Transfusion profile** |  |  |  |
| Preoperative transfusion, n (%) | 6 (20.0) | 108 (10.9) | 0.135 |
| Intraoperative transfusion, n (%) | 3 (10.0) | 188 (19.0) | 0.247 |
| Postoperative transfusion, n (%) | 15 (50.0) | 420 (42.4) | 0.456 |
| Total volume of perioperative transfusion, pack (range) | 1.5 (1.8) | 1.7 (2.7) | 0.758 |
| **Complications, n (%)** |  |  |  |
| Pneumonia | 6 (20.0) | 54 (5.4) | **0.006** |
| DVT | 0 (0) | 17 (1.7) | 1.000 |
| PTE | 0 (0) | 11 (1.1) | 1.000 |
| MI/Angina | 2 (6.7) | 18 (1.8) | 0.114 |
| UTI | 0 (0) | 47 (4.7) | 0.394 |
| Acute PJI | 1 (3.3) | 15 (1.5) | 0.382 |
| **Reoperation rate due to any reason, n (%)** | 0 (0) | 57 (5.8) | 0.406 |
| **Hospital stay, days (SD)** | 18.0 (16.8) | 13.0 (11.1) | 0.118 |
| **ICU admission, n (%)** | 5 (16.7) | 145 (14.6) | 0.792 |
| **Mortality rate,** **n (%)** |  |  |  |
| Death during the admission period | 0 (0) | 5 (0.5) | 1.000 |
| 30-day mortality | 1 (3.3) | 14 (1.4) | 0.369 |
| 1-year mortality | 12 (40) | 86 (8.7) | **<0.001** |

DVT; deep vein thrombosis; ICU, intensive care unit; IQR, interquartile range; mHHS; modified Harris hip score; MI; myocardial infarction; Op., operation; PJI, prosthetic joint infection; PS, propensity score; PTE, pulmonary thromboembolism; SD, standard deviation; UTI, urinary tract infection

**Comparison between colorectal cancer patients and non-cancer patients**

|  | **Gastric Ca.** (n=31) | **Control** (n=991) | **P-value** |
| --- | --- | --- | --- |
| **Operative time, min (SD)** | 63.8 (28.0) | 66.6 (27.8) | 0.579 |
| **Transfusion profile** |  |  |  |
| Preoperative transfusion, n (%) | 6 (19.4) | 108 (10.9) | 0.146 |
| Intraoperative transfusion, n (%) | 2 (6.5) | 188 (19.0) | 0.099 |
| Postoperative transfusion, n (%) | 11 (35.5) | 420 (42.4) | 0.468 |
| Total volume of perioperative transfusion, pack (range) | 1.6 (2.4) | 1.7 (2.7) | 0.931 |
| **Complications, n (%)** |  |  |  |
| Pneumonia | 3 (9.7) | 54 (5.4) | 0.247 |
| DVT | 1 (3.2) | 17 (1.7) | 0.428 |
| PTE | 0 (0) | 11 (1.1) | 1.000 |
| MI/Angina | 0 (0) | 18 (1.8) | 1.000 |
| UTI | 2 (6.5) | 47 (4.7) | 0.657 |
| Acute PJI | 0 (0) | 15 (1.5) | 1.000 |
| **Reoperation rate due to any reason, n (%)** | 2 (6.5) | 57 (5.8) | 0.698 |
| **Hospital stay, days (SD)** | 13.6 (14.9) | 13.0 (11.1) | 0.757 |
| **ICU admission, n (%)** | 4 (12.9) | 145 (14.6) | 1.000 |
| **Mortality rate,** **n (%)** |  |  |  |
| Death during the admission period | 0 (0) | 5 (0.5) | 1.000 |
| 30-day mortality | 0 (0) | 14 (1.4) | 1.000 |
| 1-year mortality | 4 (12.9) | 86 (8.7) | 0.757 |

DVT; deep vein thrombosis; ICU, intensive care unit; IQR, interquartile range; mHHS; modified Harris hip score; MI; myocardial infarction; Op., operation; PJI, prosthetic joint infection; PS, propensity score; PTE, pulmonary thromboembolism; SD, standard deviation; UTI, urinary tract infection
